# Genomic approaches to coronary artery disease

**Published:** 2010-11

**Authors:** Sandosh Padmanabhan, Claire Hastie, Dorairaj Prabhakaran, Anna F. Dominczak

**Affiliations:** *Institute of Cardiovascular and Medical Sciences, University of Glasgow, Glasgow, United Kingdom*; * *Centre for Chronic Disease Control, New Delhi, India*

**Keywords:** Candidate gene, coronary artery disease, genetic associations, metabolomics, proteomics

## Abstract

Coronary artery disease (CAD) is a leading cause of death and disability worldwide. In addition to lifestyle and environmental factors which are major aetiologic determinants, there is considerable familial clustering of the disease indicating a genetic component in its causation. Although the total genetic contribution to CAD risk can be quantified, the determination of the size and number of contributing effects is impossible without identifying all CAD susceptibility genes. However, despite extensive studies, strong evidence of a molecular genetic association with coronary artery disease or myocardial infarction remains elusive. Genome wide association studies have been successful in identifying robust associations of single nucleotide polymorphisms (SNP) with CAD. Identifying the causal variant and dissecting pathways linking these variants to disease process is a major challenge. Technologies from whole genome sequencing, proteomics, transcriptomics and metabolomics are now available to extend analysis to a more complete range of potential susceptibility variants, and to support more explicit modelling of the joint effects of genes and environment. The availability of these high throughput technologies does not diminish the importance of rigorous phenotyping and appropriate study designs in all the endeavours to understand the aetiopathogenesis of CAD. Combining classical epidemiology with modern genomics will require collaborative efforts within the cardiovascular disease community at both bench and bedside and this will have the potential to expand our understanding of CAD and translate discoveries into clinically useful applications that will have a major impact on public health.

## Introduction

Coronary arery disease (CAD) is predicted to be the most common cause of death globally, including India, by 2020, when the 10 leading causes of disability-adjusted life-years (in descending order) are projected to be ischaemic heart disease, unipolar major depression, road traffic accidents, cerebrovascular disease, chronic obstructive pulmonary disease, lower respiratory infections, tuberculosis, war injuries, diarrhoeal diseases, and HIV^1^,^2^. In India, this epidemiological transition is attributed to recent rapid economic and social changes leading to non-communicable diseases attaining the top spot for causing death and disability, and chronic diseases contributing to an estimated 53 per cent of deaths and 44 per cent of disability-adjusted life-years lost^1^,^3^. By the year 2010, it is projected that 60 per cent of the world’s patients with heart disease will be in India^2^. CAD also remains the number one cause of death in industrialised countries despite collective efforts to minimize attributable risk from known contributors to CAD such as hypertension, dyslipidaemia and smoking. The proportion of deaths due to non-communicable disease is projected to rise from 59 per cent in 2002 to 69 per cent in 2030^4^. The epidemiological transition needs not only country-wide health planning and preventive strategies, but also national collaborative research into molecular and genetic causation of these diseases in India. Advances from cardiovascular research in genetic studies, expression profiling and proteomics will not reach their full potential unless translated to care of the individual patient, and this requires combining new molecular discoveries with epidemiological and screening tools. The focus of this review is to first present the evidence for a genetic component to coronary artery disease then, describe the recent progress in genomic and other high throughput technologies in understanding the causation of atherosclerotic cardiovascular disease, and finally present the need for further research requiring the collaboration between clinicians, basic scientists and epidemiologists (Fig.).

**Fig:**
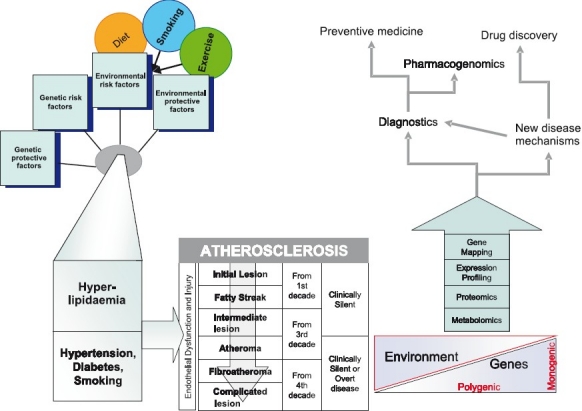
Integrating epidemiological and “Omics” strategies in a translational framework for coronary artery disease.

## Genetic Basis of CAD

CAD comprises a spectrum of clinical diagnoses [*e.g*. angina, myocardial infarction (MI)] which are caused by atherosclerosis, a pervasive degenerative condition in which lipid and fibrous matrix is deposited in arterial vessel walls to form atheromatous plaques. Rupture of unstable plaques in coronary arteries results in the release of thrombogenic material into the lumen of the vessel leading to coronary thrombosis, vessel occlusion, and subsequent infarction of the myocardium, a critical condition with high mortality^5^.

### 

#### Familial clustering

The key factor that indicates the presence of a genetic component to atherosclerosis is the familial clustering of CAD^6^,^7^. The strength of the association between parental history and CAD is similar to that found for other standard risk factors such as systolic BP, cholesterol and cigarette smoking. A history of early CAD in a first degree relative approximately doubles the risk of CAD (RR 1.3-11.3)^6^,^8^,^9^. In the Framingham Heart Study, parental history of premature coronary, cerebral vascular or peripheral vascular disease was a strong risk factor for CAD (OR=2.0 for men and 1.7 women) after adjustment for all classical risk factors^7^. In the INTERHEART study, family history of CAD after adjustment for nine classical risk factors was associated with an OR of 1.45^9^. Additionally, there is increasing evidence that some of the classical risk factors for atherosclerosis also have independent genetic components [lipids, BMI, BP, lipoprotein(a), homocysteine, type 2 diabetes, fibrinogen, C-reactive protein (CRP)]^10^. The extent to which the familial clustering of CAD can be explained by heritable quantitative variation in the classical risk factors such as cholesterol and BP is contentious, and the small but significant residual familial relative risk after regression on these conventional risk factors suggests the presence of unmeasured genetic risk factors, raising the possibility of a high level of genetic control by a small number of genes that control fundamental physiological systems^6^,^7^,^9^,^11^.

#### Heritability

A 36 year follow up study of 21000 Swedish twins demonstrated concordance rates for acute myocardial infarction in monozygotic (MZ) twins of 0.26 among males and 0.27 among females. For dizygotic (DZ) twins the concordances were 0.20 for males and 0.16 for females^12^. The estimated heritability was 57 per cent among male twins and 38 per cent for female twins for CAD^13^. Such high heritabilities are supported by adoption studies showing a relative ris k of death in adoptees of 4.5 for death from vascular disease before the age of 50 yr of a biological parent compared to a relative risk of 3.0 for death of an adoptive parent^14^. Further evidence for a moderate genetic influence on the risk of dying prematurely in adulthood, with only a small effect of the family environment was shown by higher mortality among biological parents who had children dying in the age range 25 through 64 yr for death from natural causes, infectious causes, vascular causes, and from all causes combined, with no significant effects for the adoptive parents^15^.

#### Mendelian forms of CAD

Studies of rare Mendelian forms of CAD have shown how mutations in genes [low density lipoprotein receptor (LDLR), apolipoprotein B (ApoB), proprotein convertase subtilisin/kexin type 9 (PCSK9), low density lipoprotein receptor adaptor protein 1 (ARH), ATP-binding cassette, sub-family G (WHITE), member 5 (ABCG5), cytochrome P450, family 7, subfamily A, polypeptide 1 (CYP7A1)]^10^ involved in low-density lipoprotein and high-density lipoprotein metabolism/homeostasis can cause premature CAD, but these mutations are thought to explain a relatively minor fraction of familial CAD. However studies of monogenic diseases have helped to unravel the molecular pathways that regulate cholesterol metabolism, resulting in the development of HMG-CoA-reductase inhibitors^16^. Also, studies of two rare genetic lipid disorders, Tangier disease and sitosterolemia, have revolutionized our understanding of sterol transport^17^,^18^.

All these point to a multifactorial polygenic causation of CAD, with evidence that susceptibility genes for CAD might exist that are independent of hypercholesterolaemia or arterial hypertension or diabetes and may act by affecting an intermediate factor such as vascular inflammation, oxidative stress, thrombogenicity, arterial calcification, *etc*. It is likely that susceptibility genes might be involved in novel biological pathways or be novel genes in well known pathways such as cholesterol metabolism. The search and identification of genetic factors contributing to CAD is important to increase our understanding of CAD pathophysiology and develop new strategies for risk prediction and intervention.

## Genetic Architecture of CAD

The success of CAD gene mapping is dependent on its genetic architecture which refers to the number of disease genes that exist, their allele frequencies, the risks that they confer, and the interactions between multiple genetic and environmental factors^19^,^20^. Although the total genetic contribution to CAD risk can be quantified, the determination of the size and number of contributing effects is impossible without identifying all CAD susceptibility genes. The multiple risk factors for CAD themselves have their own genetic architecture. The heritabilities of some of the risk factors for CAD are considerable - total cholesterol (40 to 60%), HDL-cholesterol (45 to 75%), total triglycerides (40 to 80%), body mass index (25 to 60%), systolic blood pressure (50 to 70%), Lp(a) levels (90%), homocysteine levels (45%), type 2 diabetes (40 to 80%), fibrinogen (20 to 50%)^10^. Also, as CAD is rare before the age of 50 yr, it is unlikely to have an effect on reproductive success and hence less likely to have been subject to direct evolutionary selection pressure. Variants that confer susceptibility or protection for CAD might therefore have evolved neutrally in the past, and so could present at a wide range of frequencies. This is the basis of the Common Disease/Common Variant (CDCV) hypothesis which holds that the genetic variants underlying complex traits occur with a relatively high frequency (>1%), have undergone little or no selection in earlier populations and are likely to date back to >100,000 years ago^21^.The other competing model is the Common Disease Rare Variant hypothesis, with an inverse relationship between the magnitude of genetic effect and allele frequency^22^. This model argues that diseases are common because of highly prevalent environmental influences, not because of common disease alleles in the population^19^. A review of candidate gene associations and recent genome wide association study results support the importance of common alleles in CAD^23^,^24^. At odds with this, rare allelic variants of three candidate genes (*ABCA1, APOA1, LCAT*) that influence HDL levels, were jointly found to make a substantial contribution to the population distribution of HDL levels^25^,^26^. The most likely scenario would be that the allelic spectrum of the disease variants is the same as the general spectrum of all disease variants. Under this neutral model, although most susceptibility variants are rare with minor allele frequencies (MAF) <1 per cent, variants with MAF>1 per cent would account for more than 90 per cent of the genetic differences between individuals. It is plausible that these common variants might contribute significantly to those common diseases in which susceptibility alleles might not be under intense negative selection.

## Subject ascertainment and study designs

### 

The common methods used in the genetic dissection of complex diseases like CAD are linkage and association studies. Linkage mapping (a gene hunting technique that attempts to identify gene regions that are found in people with disease more frequently than in those without the disease, by studying genetic markers of known chromosomal location that are co-inherited with the disease in a pedigree), which is a powerful tool for finding Mendelian disease genes, often produces weak, and sometimes inconsistent, signals in complex disease studies^27^. Association mapping is another study design commonly used in candidate gene studies and is based on linkage disequilibrium. Linkage disequilibrium (LD) is the non-random association of alleles at two or more loci on a chromosome and results in the greater co-occurrence of two genetic markers on the same chromosome in a population than would be expected for independent markers. To succeed in finding complex disease genes, a study must detect a relatively weak statistical signal, and hence study design flaws can potentially have a dramatic impact on the probability of success^28^.

Ascertainment criteria partitioning the presence or absence of CAD are a major challenge and have major implications on the power and success of gene mapping studies. Studies of asymptomatic individuals have shown that atherosclerotic CAD is pervasive, but only a fraction of them will ever develop an MI, and this is influenced by haemodynamic modifiers or individual susceptibility to inflammation and plaque progression^29^,^30^.

#### Intermediate phenotypes

Many of the known risk factors for the development of CAD are genetically determined traits - LDL and HDL levels, high blood pressure, type 2 diabetes and metabolic syndrome. A growing number of other plasma factors have also been identified that are associated with CAD risk, many of which are also strongly genetically determined [Lp(a), homocysteine and fibrinogen]^10^. Combined genetic and intermediate analysis with single, large study groups provides a means of comparing the risk that is attributable to genetically determined life-long variations in the measured intermediate phenotype with the expected increment in CAD risk that is based on observational epidemiological studies^5^. This approach, sometimes referred to as “Mendelian randomisation”, could potentially discriminate between causal relationships and those that are due to confounding or “reverse causality”^31^. Component phenotypes of atherosclerosis can be expected to reflect the influence of a smaller number of genes that should therefore have a larger effect size and increased study power. These phenotypes also provide surrogate endpoints in longitudinal studies and allow the classification of patients into more homogeneous groups. Examples of quantitative vascular phenotypes include carotid intima-medial thickness, non invasive measures of endothelial function or arterial stiffness^5^.

#### Cases

The selection of cases or ‘affected’ takes into consideration not only phenotypic definition to reduce phenotypic heterogeneity, but also manoeuvres to improve study power through enrichment of specific disease-predisposing alleles. There is an argument for using related cases, as they are more likely to share a disease-causing variant in common than randomly selected cases, provided the data are analyzed after taking into account correlation between related individuals^32^,^33^. Individuals with CAD who have low risk profiles in terms of lifestyle factors will carry greater genetic loads and thus be more informative for genetic studies. One strategy adopted by researchers is to bias their recruitment strategy towards younger patients. For the myocardial infarction phenotype prematurity refers to age less than 45 for men or less than 50 yr for women for the index event. However, it is important to note that in India about 50 per cent of CHD-related deaths occur in people younger than 70 yr compared with only 22 per cent in the West^2^. Genetic enrichment can also be achieved by enrolling individuals with first degree relatives who are affected as cases^34^.

There are important factors to be considered when selecting a CAD phenotype for study^34^. It is important to note that myocardial infarction represents a very small fraction of the individuals who have the CAD phenotype. A large proportion of people with CAD do not have anginal symptoms and are identified only through an abnormal routine exercise test. Patients who present for a coronary angiogram represent a bias of ascertainment. Also the coronary angiogram is an incomplete assessment which only shows the accumulation of plaque that results in narrowing of the arterial lumen. The complexity increases further when one considers multiple longitudinal angiographic studies that show an individual progressing from having slight (<30%) narrowing to critical (>70%) stenosis over a 6 month period. Thus it is important to acknowledge that the assessment of a “case” is only relevant to the actual time in which the study was performed and that this is a dynamic phenomenon. Safeguards are therefore needed to pre-empt the potential re-categorisation of a control to a case, and ensure that the defined phenotype is homogeneous as there are vast pathophysiological differences between the chronic accumulation of arterial plaque as compared with a sudden plaque rupture event with attendant thrombosis. The phenotype of myocardial infarction, one of the manifestations of CAD, is more restrictive and has thus far proven more useful in identifying susceptibility genes. However, the diagnosis of MI does not imply a uniform pathogenic process. A significant minority of patients with MI present with sudden cardiac death and never reach the hospital, leading to a survival bias. Also an even larger proportion of patients have an acute coronary syndrome with a normal ECG or non specific ECG abnormalities. There are important differences between ST segment elevation myocardial infarction which involves occlusive coronary thrombosis with more extensive myocardial necrosis, and non-ST segment elevation myocardial infarction which occurs with non-occlusive thrombosis and may result from more extensive collateral flow and results in less necrosis. These phenotypic differences may be associated with differences in genetic predisposition.

#### Controls

The definition of controls or ‘unaffected’ is more challenging than cases^34^,^35^. An ideal control might be conceived as an individual who has lived to greater than 90 yr and has undergone a post-mortem that shows completely normal coronary arteries, and the issues in addition to impracticality are bias introduced in terms of longevity of the controls and the different diagnostic criteria between cases and controls.

From a practical point of view, it would be ideal to identify controls without having to subject individuals to an invasive assessment. However, stress testing, even with adjunctive nuclear or echocardiographic imaging is not sensitive. Currently the gold standard technique is an invasive diagnostic angiogram which is expensive and carries a definite albeit low risk of morbidity and death. The definition of a normal angiogram is still unclear, and the appearance of a truly “normal” angiogram is not particularly common as compared with finding individuals who have minor irregularities with no frank luminal encroachment that would approximate a 10 per cent narrowing^34^. Many of the patients with true “normal” angiograms have actually presented to cardiac catheterisation for evaluation of valvular heart disease, thus introducing a confounding factor. Also a normal angiographic study does not exclude small vessel disease, and it would be inappropriate to classify them as controls. It is also important that controls should not have other forms of atherosclerotic disease such as stroke or transient ischaemic attacks or peripheral vascular disease. One of the important considerations is matching cases and controls. There exists the distinct possibility that controls may, later in life, become cases. For reasons of genetic enrichment as described above, young cases are likely to be preferentially collected. But comparison of young cases (who are less than 55 yr old) with aged (greater than 85 yr old) controls introduces potential confounding sources. Examples of these are differential survival due to genes that are unrelated to CAD, genetic drift and mismatching for potential covariates^5^.

#### Case-control, cohort or family designs

Using a positional cloning approach, linkage analysis using families of multiple affected individuals was very successful in identifying genes for Mendelian diseases. However, it has been less successful in identifying genes for complex diseases. Recently genome wide association studies which have been successful in identifying replicated loci for common diseases have predominantly used a case-control approach, where samples of unrelated affected and unaffected individuals are ascertained from the study population. While it is extremely easy to recruit unrelated cases and control, recruitment of families is resource intensive. But it must be recognised that population and family studies have different strengths and weaknesses and must be viewed as complementary and not competitive^35^,^36^.

A key concern with case-control design is latent population substructure that will inflate type I error and care must be taken when attempting to reproduce these results in other populations. Though there are statistical methods to correct for population substructure along with information from ancestry informative markers, these are only applicable to populations of European ancestry, these will correct only for ‘average’ genome wide measures of ethnic admixture and will not always eliminate spurious associations immediately adjacent to markers that are strongly informative about ancestry^35^,^37^–^39^. There is clear evidence that analysis in African-descent populations is complicated by their greater haplotypic diversity and fine-scale geographical structure^40^. Recent papers have demonstrated the difficulty of genetic analysis in the Hispanic or Latino populations due to subgroups with various amounts of contribution from ancestral Native-American, West African and European populations and the Wellcome Trust genome wide association (GWA) identified a substructure within the European-Caucasian population even within the UK^24^,^35^,^41^–^43^.

The key advantage of family studies is that they are robust to population stratification^36^. Though family based design is associated with some loss of power, in populations with greater diversity and substructure, this is more advantageous. The additional benefits of family information are that it helps to refine the genetic model and risk estimates, control for the effects of shared environment and detect heterogeneity, imprinting, and epigenetic phenomena^36^,^44^. Finally if the underlying architecture favours the rare variant hypothesis, then one can expect extensive allelic and locus heterogeneity, and in this case family studies are preferred to case control studies^44^. One option for the efficient use of family data in such a setting is to restrict high-density scanning to a subset of pedigree members and then use information on patterns of chromosomal segregation derived from low-density genotyping in the remaining members to propagate genotypes through the family^45^.

The successful detection of gene-gene or gene-environment interactions is dependent on recruiting adequate sample sizes in the prospective cohort studies. In contrast to case-control studies which typically begin when disease cases have occurred, prospective cohort studies avoid this inherent bias by investigating a representative sample of the population before disease onset. In terms of genetic main effects, 5000 incident cases are needed for a minimum detectable odds ratio (MDOR) of 1.5 with 80 per cent power and MAF=5 per cent. For genotypic and environmental prevalences of 10 per cent and above, 10,000 cases are needed to provide adequate power for interaction effects with an MDOR greater than 2^35^,^46^,^47^. These studies promise new insights into the genetic basis of continuous traits and enhanced opportunities for revealing pleiotropy, although low power remains an issue - especially for the detection of non-additive gene-environment interactions^46^,^47^.

## Linkage Studies

Previous methods for determining genetic linkage of a complex disease relied chiefly upon the technique of genome wide scanning of microsatellites (short tandem repeat sequences). This required the laborious selection of hundreds of families, particularly sib pairs with MI or CAD. Using around 400 microsatellite markers distributed evenly across the genome, the goal was to identify a significant linkage peak defined by a logarithm of odds ratio (LOD) greater than 3.5 corresponding to a p<10^-6^ indicating a gene that is in linkage disequilibrium (LD) near or even within the microsatellite region. However, identifying disease causing genes interspersed within microsatellites has proved to be quite difficult and studies from different centres reported varying results - Finland (2q21.2-22 and Xq23-26)^48^, Germany (14q32.2)^49^, Iceland (13q12-13)^50^, US (1p34-36,3q13 and 5q31)^51^ and UK (2p11, 17p11-17q21)^52^,^53^. Genomic regions identified in the published linkage studies as being correlated with CHF are largely non-overlapping, suggesting genetic and/or phenotypic heterogeneity. Two genes, *ALOX5AP* and *MEF2A*, have been identified by fine mapping studies following the original linkage analysis. The Icelandic locus was replicated in population-based studies from Iceland and England, with different haplotypes of the *ALOX5AP* gene (encoding 5-lipoxygenase activating protein) associated with CAD in the two countries, and the Icelandic haplotype was also associated with stroke in Iceland and in Scotland^50^,^54^. MEF2A (myocyte enhancer factor 2A) a transcription factor expressed in coronary artery endothelium was identified by linkage analysis in a pedigree in which 13 members had CAD, nine of whom had MI^55^.

## Candidate genes studies

Candidate gene association studies often base selection of candidate genes on assumptions about biologically relevant genes, and hence these studies are biased against identification of novel genes. An overlap in inflammatory responses observed in chronic infection and atherosclerosis led to an interest in candidate genes for CAD that are involved in innate immunity^56^. Among these, CD14, toll-like receptor 4 (TLR 4), were the first genes to show variable evidence of association with CAD susceptibility^57^–^59^. However, large association studies showed no association with susceptibility^60^. Leukotriene A4 hydrolase (LTA4H) has also been shown to be associated with CAD, with striking evidence in African American patients and modest association among Europeans^61^.

In a pioneering study^62^ of nearly 93,000 predominantly gene based SNPs in a Japanese case control cohort, polymorphisms in the lymphotoxin-α (LTA) gene, which encodes a member of the tumour necrosis factor (TNF) ligand family were found to be associated with susceptibility to myocardial infarction. A larger case-control study however did not find any association^63^.

Despite some successes, single gene association studies remain problematic, with most being poorly replicable^23^,^64^. Because only a small proportion of variants will have significant effect sizes, these studies have a low *a priori* likelihood of identifying the important variants. However, as small scale studies are easy to carry out these have become numerous; some, by chance, yield a false positive association and there is evidence of publication bias in the literature that might enrich for false positives.

## Genome Wide Association (GWA)

Of the 3.1 billion base pairs in our genome, our inter-individuality is determined by only 0.1 per cent or approximately 3 million of those base pairs. GWA studies are large scale association mapping using SNPs, making no assumptions of the genomic location or function of the causal variant and are a comprehensive approach to testing the hypothesis that common alleles contribute to heritable phenotype variation^33^. Because no assumptions are made about the genomic location of the causal variants, this approach could exploit the strengths of association studies without having to guess the identity of the causal genes. The GWA approach, therefore, represents an unbiased yet fairly comprehensive option that can be attempted even in the absence of convincing evidence regarding function or location of the causal genes. The crucial factors that have made GWA studies possible are the availability of high throughput technologies, the decreasing cost of genotyping and the determination of LD on a genome wide scale through the HapMap project^40^. The recent surge in GWA studies for complex diseases, including CAD, with replicated results of SNP association show that complex diseases are finally yielding their secrets^24^,^65^–^69^. Most of these studies are case-control studies, where SNP frequencies are compared between the two groups, and those that differ significantly are then validated in independent samples. Most of these robust associations have not been with genes previously suspected as being related to the disease. Some of these associations have been found in regions not even known to harbour genes. Such findings promise to open up new avenues of research, through both the discovery of new genes relevant to specific diseases and the elucidation of genetic mechanisms.

The association of 9p21 locus with CAD and MI has been found in every GWA studies published including a meta-analysis which suggests, at least, that this association is widely distributed^66^,^70^–^72^. However, the region on chromosome 9p21 associated with CAD is defined by two flanking recombination hotspots and contains the coding sequences of genes *CDKN2A* and *CDKN2B*. Thus a large number of SNPs in this region show highly significant association with CAD and the causally responsible variant and the related mechanism remains to be identified. The *CDKN2A* and *CDKN2B* genes are cyclin-dependent kinase inhibitors which regulate cell cycle and belong to a family of genes that have been implicated in the pathogenesis of atherosclerosis through their role in transforming growth factor-β-induced growth inhibition. However, the most strongly associated SNPs lie considerably upstream of these genes, and the nearest signal is 10 kb upstream of *CDKN2B*. The C-allele of the lead SNP (rs1333049) has a frequency of 0.17 in Yoruba, 0.48 in Han Chinese, 0.51 in Japanese and 0.49 in Europeans indicating that the risk of CAD and MI related to the chromosome 9p21 locus may vary among different ethnic groups. More interestingly, this same susceptibility locus was found to be independently associated with type 2 diabetes^67^–^69^,^73^. It is possible that the risk allele is located within a regulatory element that controls the expression of a gene outside of the associated region, or that the functional variant itself is located outside the region that was not well covered by the genotyping platforms used in the studies. Other genes associated with significantly increased risk of MI are proline/serine-rich coiled-coil 1 (*PSRC1*), melanoma inhibitory activity family, member 3 (*MIA3*) and mothers against decapentaplegic homolog 3 also known as SMAD family member 3 (*SMAD3*) encoding cell-growth regulators, and a locus near the gene encoding the chemokine (C-X-C motif) ligand 12 (*CXCL12*)^71^.

There is also evidence of strong signals for intermediate phenotypes from GWA studies. The Diabetes Genetics Initiative was designed to investigate type 2 diabetes, but 18 other phenotypes, including plasma lipid concentrations, were analysed as secondary traits^67^. Several of the loci that are significantly associated with changes in lipid concentrations are in or near genes in which mutations have been shown to cause mendelian syndromes affecting lipid concentrations [namely genes that encode the proteins apolipoprotein E, ABCA1, apolipoprotein A-V, Cholesterylester transfer protein (*CETP*), lipoprotein lipase and hepatic lipase]. In addition, a SNP in the gene glucokinase (hexokinase 4) regulator (*GCKR*), which encodes glucokinase regulatory protein, was found to have a highly significant association with triglyceride concentrations^67^. Other loci identified are near methylmalonic aciduria type B1/mevalonate kinase (*MVK-MMAB*) and UDP acetylgalactosaminyltransferase 2 (*GALNT2*), with high-density lipoprotein (HDL) cholesterol; near sortilin 1 (*SORT1*), cadherin EGF LAG seven-pass G-type receptor 2 (*CELSR2*) and Proline/serine-rich coiled-coil protein 1 (*PSRC1*) with variants primarily associated with low-density lipoprotein (LDL) cholesterol; near Tribbles homolog 1 (*TRIB1*), MLX interacting protein-like1 (*MLXIPL*) and angiopoietin-like 3 (*ANGPTL3*), with variants primarily associated with triglycerides; and a locus encompassing several genes near neurocan (*NCAN*), with variants strongly associated with both triglycerides and LDL cholesterol^74^,^75^.

For most phenotypes, genes that were previously identified by linkage analysis, however, have not been found to be associated using GWA studies. There are several explanations for this- the particular chip configuration used covered these genes poorly and so the genetic effect was missed; the quantitative contribution of the genes to the disease is lower when ranked compared with other genes; or the association was missed because it was not present to a great degree in a particular study sample^35^,^42^,^43^. There is always the possibility that results from GWA studies are merely random because subject recruitment is in fact sampling from a population and the results are due to statistical methods.

## Resequencing and other ‘Omic’ approaches

### 

Validating new discoveries related to atherosclerosis will require multiple complementary approaches.

#### Resequencing

The GWA approach has been extremely successful in identifying common SNP variants with modest effect on the CAD phenotype. However, this does not detect whether rarer variants play a role in CAD and it is unable to explain all of the observed familial clustering. All these emphasize the need to extend analysis to a more complete range of potential susceptibility variants, and to support more explicit modelling of the joint effects of genes and environment^35^. Also, progressing from confirmed association signal to complete enumeration of the pattern of causal variants at a given locus poses significant challenges, and to narrow down the causal variant will require fine-mapping and resequencing of the implicated region. For example, sequence variants in the *PCSK9* gene were associated with lower LDL cholesterol and protection from CVD, single gene mutations in sarcomere genes were associated with a small but significant proportion of men and women with left ventricular hypertrophy (LVH)^76^,^77^. Whole-genome resequencing^78^ would resolve the question regarding the relative contribution of rare and common variants to CAD, but at present whole-genome resequencing is prohibitively expensive. An alternative option is to study different ethnicities as inter-ethnic differences in patterns of LD and mutations are extremely valuable tools for fine mapping.

#### Transcriptomics

The SNP association studies cannot determine which SNPs may affect gene expression or function. Gene expression microarray technology provides a way to scan rapidly specific tissues for patterns of expression among thousands of genes across the genome and provides both qualitative (switched on/off genes) and quantitative data (transcriptional level of single genes), so that subtle differences of gene activation can be detected^79^. Although SNPs represent static information (the genome), expression profiles are dynamic (the transcriptome) and may show physiological fluctuations. Nevertheless, profiling cardiovascular tissue samples may generate novel hypotheses and help to identify unexpected cell components and reveal novel marker genes and may aid identification of drug targets and validation of candidate drugs for the management of atherosclerosis. As proof of principle, Tuomisto *et al*^80^ showed that HMG-CoA reductase (the target of statin drugs) increased in atherosclerotic plaque. Studying the transcriptional effect of statin exposure on peripheral monocytes, Waehre *et al*^81^ provided evidence showing that statins inhibit expression of inflammatory cytokine interleukin-1β, normally present at high levels among subjects with CAD.

#### Proteomics

There are approximately ten times more proteins than genes, and expression arrays should preferably be complemented by proteomic evaluation as they cannot assess potentially important post-transcriptional variables including alternative splicing of mRNA, control subjects on protein translation, and post-translational processing of proteins^82^,^83^. Therefore, new findings discovered by transcript profiling may serve as leads but require subsequent functional characterization. Proteomic studies on isolated plasma lipid fractions have yielded new insight into the composition of LDL and high-density lipoprotein particles, identifying 3 proteins previously not associated with LDL and 2 proteins not previously associated with high-density lipoprotein and identifying unique patterns of LDL associated apolipoproteins in subjects with type 2 diabetes and subclinical peripheral atherosclerosis^84^–^86^. Urinary proteomic studies are also useful and have been shown to identify CAD patients and help in monitoring the effects of therapeutic interventions^87^. As in the case of genomic markers, proteomic studies require rigorous validation of technology platforms and experimental results^88^.

#### Metabolomics

The study of the collection of small molecular-weight organic and inorganic species present in a biological system is defined as metabolomics, and provides a phenotypic profile either as a snapshot in time or as an integrated picture of biology over a period of time. Metabolites are the final downstream product of gene transcription and so reflect more closely the phenotype at a functional level, and the metabolome is thought to be a potentially more sensitive marker of cellular processes both in normal physiology and disease.

As proof of principle, Sabatine *et al*^89^ used mass spectrometry-based technology to identify differences in plasma metabolites among 18 subjects with ischaemia induced by exercise stress testing compared with non ischaemic individuals who also exercised; specifically, changes in 6 metabolites, including citric acid, accurately differentiated cases from control subjects (*P*<0.0001). Analysing 4,630 participants from the INTERMAP epidemiological study, involving 17 population samples aged 40-59 in China, Japan, UK and USA, Holmes *et al*^90^ showed that urinary metabolite excretion patterns for East Asian and western population samples, with contrasting diets, diet-related major risk factors, and coronary heart disease/stroke rates were significantly differentiated (*P*<10^-16^) and mean 24-hour urinary excretion of alanine (direct) and hippurate (inverse), reflecting diet and gut microbial activities, were also associated with blood pressure of these individuals.

Thus metabolomic analysis may not only identify potential biomarkers for CV disease but may also provide information regarding prognosis, response to therapy, and underlying mechanisms of the disease.

## Conclusions

Rapid advances in genomic, proteomic, and metabolomic technology provide researchers with valuable tools for understanding the genetic predisposition to atherosclerotic cardiovascular disease and have led to the development of successful therapeutic interventions for atherosclerotic cardiovascular disease; for example, reducing plasma concentrations of atherogenic lipoproteins (with statins) and blocking rennin-angiotensin-aldosterone system activity (with ACE inhibitors and angiotensin-receptor blockers). Combining classical epidemiology with modern genomics will require collaborative efforts within the cardiovascular disease community at both bench and bedside, and is likely to pay major dividends with the development of a new generation of personalised prevention and treatment interventions that will have a major impact on public health.
